# Health system strengthening and the importance of public investment: a study of National Rural Health Mission in Bihar

**DOI:** 10.1186/1753-6561-6-S5-O11

**Published:** 2012-09-28

**Authors:** Indranil Mukhopadhyay

**Affiliations:** 1 Public Health Foundation of India, New Delhi, India

## Introduction

Chronic under-investment in government health systems has led to significant weakening of these systems and crippled their ability to deliver good quality health care. Through the introduction of the National Rural Health Mission (NRHM), there have been efforts to increase public spending in health and strengthen health systems in rural areas. However, evidence shows that the increase in fund did not necessarily result in its utilisation. In the initial period of NRHM, a considerable amount of funds were under-utilised. It was argued that further increase in spending is unwarranted as the public health system does not have the capacity to absorb funds.

The present study is an attempt to draw linkages between system strengthening and fund absorption capacity to argue that only consistent investment can make health systems stronger. This in turn would allow the system to absorb more funds. We tracked the fund flow and fund absorptive capacity under the NRHM at various levels: state, district, block and village. We sought to understand reasons for under-spending and study the various aspects of quality of spending in a district in Bihar.

## Methods

Public expenditure data obtained from various levels were analysed. Document analysis of Financial and Management Reports, Audit Reports, Utilisation Certificates was undertaken. Select key informant interviews of stakeholders were conducted using a semi-structured questionnaire to understand causes, processes, institutional practices and organizational culture effecting fund utilization. The data were analysed in order to get an overall picture of the nature and trend of resource allocation vis-a-vis utilisation at state and district levels.

## Results

Public investment in health is among the lowest in India, when measured in terms of share in Gross Domestic Product. As the results of the study show, since the introduction of NRHM, there has been some growth in the funds disbursed to the states. But, the increase in spending at the state, district and block levels was not adequate, leaving huge unspent balances.

The quarterly break-up of fund flow shows that a bulk of the funds reached the districts only during the latter part of the financial year, hampering the ability to spend. A major bottleneck was the issue of capacity building of staff to deal with the increase in level of spending and comprehending the advance guidelines of the NRHM.

Compared to previous years (before the National Rural Health Mission) fund utilisation has increased and quality of spending has improved. Fund flow processes have become more efficient. Fund absorptive capacity has relatively increased compared to last few years. However, utilisation had been more in activities in the form of entitlements like cash incentives for promoting family planning, safe motherhood programme or activities that run on a vertical mode like the Pulse Polio Immunisation. Funds for activities that required innovation remained under-utilised.

## Discussion

Improving absorptive capacity of funds by the states is a long-term process. It would require sustained efforts towards strengthening management and institutional capacities, filling up of vacant posts, higher salaries, greater expenditure on drugs and other consumables.

The task of health systems strengthening has gained momentum over the last two-three years through the NRHM. A rearrangement of the financial mechanism with greater share of states and much greater emphasis on decentralisation may be pointers for a future roadmap on the government’s health interventions in the country. NRHM is at best a small step forward in the endeavour to guarantee universal access to health. A gradual increase in health spending with long-term perspective would be crucial to supplement the measures initiated through this umbrella health intervention programme.

## Competing interests

Author declares that the study was part of research work undertaken under the Save the Children. However, the opinions expressed are author’s personal.

## Funding statement

The study was funded by the Save the Children.

